# Disruption of Abcc6 Transporter in Zebrafish Causes Ocular Calcification and Cardiac Fibrosis

**DOI:** 10.3390/ijms22010278

**Published:** 2020-12-29

**Authors:** Jianjian Sun, Peilu She, Xu Liu, Bangjun Gao, Daqin Jin, Tao P. Zhong

**Affiliations:** 1 State Key Laboratory of Genetic Engineering, School of Life Sciences, Fudan University, Shanghai 200438, China; jjsun@bio.ecnu.edu.cn; 2 Shanghai Key Laboratory of Regulatory Biology, Institute of Molecular Medicine, School of Life Sciences, East China Normal University, Shanghai 200241, China; plshe@bio.ecnu.edu.cn (P.S.); 51181300090@stu.ecnu.edu.cn (X.L.); 52181300051@stu.ecnu.edu.cn (B.G.); dqjin@bio.ecnu.edu.cn (D.J.)

**Keywords:** PXE, *abcc6*, vitamin K, ocular calcification, cardiac fibrosis

## Abstract

Pseudoxanthoma elasticum (PXE), caused by ABCC6/MRP6 mutation, is a heritable multisystem disorder in humans. The progressive clinical manifestations of PXE are accompanied by ectopic mineralization in various connective tissues. However, the pathomechanisms underlying the PXE multisystem disorder remains obscure, and effective treatment is currently available. In this study, we generated zebrafish *abcc6a* mutants using the transcription activator-like effector nuclease (TALEN) technique. In young adult zebrafish, *abcc6a* is expressed in the eyes, heart, intestine, and other tissues. *abcc6a* mutants exhibit extensive calcification in the ocular sclera and Bruch’s membrane, recapitulating part of the PXE manifestations. Mutations in *abcc6a* upregulate extracellular matrix (ECM) genes, leading to fibrotic heart with reduced cardiomyocyte number. We found that *abcc6a* mutation reduced levels of both vitamin K and pyrophosphate (PPi) in the serum and diverse tissues. Vitamin K administration increased the gamma-glutamyl carboxylated form of matrix gla protein (cMGP), alleviating ectopic calcification and fibrosis in vertebrae, eyes, and hearts. Our findings contribute to a comprehensive understanding of PXE pathophysiology from zebrafish models.

## 1. Introduction

Pseudoxanthoma elasticum (PXE; OMIM#264800) [1] is a heritable multisystem disorder that is characterized by a progressive accumulation of ectopic mineralization in connective tissues in the skin, eyes, and cardiovascular system [2,3,4,5]. PXE has a late onset, with diagnosis made at approximately 13 years of age [6]. The primary clinical manifestations of PXE include yellow papules on the skin, angioid streaks in the eyes, and ectopic mineralization in the elastic lamina of the arterial blood vessel walls. The PXE multisystem disorder progresses with severe cardiovascular disorders including hypertension, diastolic myocardial dysfunction, restrictive cardiomyopathy and coronary artery disease, leading to significant morbidity [7,8].

PXE has an autosomal recessive inheritance pattern caused by mutations in ABCC6 [9,10,11]. ABCC6 is a member of the ATP-binding cassette (ABC) transmembrane transporter subfamily C, and is also known as a multidrug resistance-associated protein (MRP) [12]. ABCC6 is expressed abundantly in the liver and kidney, with low expression in the brain, eye, heart, lung, stomach, pancreas, and gastrointestinal system in humans and mice [13,14]. Particularly, ABCC6 is detectable in a variety of cells, such as epithelial, endothelial, and smooth muscle cells, neurons, and leukocytes, reflective of its complex multifunctional function [14]. The nature of substrate transported by ABCC6 and the contributing factor to PXE remain unknown. Recent studies have reported that pyrophosphate (PPi) is an anti-mineralization factor, and reduced PPi concentrations were evident in PXE patients and *Abcc6^−/−^* mice [15]. ABCC6 mediates the release of adenosine triphosphate (ATP), converted to the mineralization inhibitor PPi and adenosine monophosphate (AMP) by ectonucleoside pyrophosphatase 1 (ENPP1) [16]. However, ABCC6 was unable to directly transport ATP in vesicular transport assays [16]. Increased PPi levels generated by ENPP1 overexpression or PPi supplementation inhibited ectopic calcification in *Abcc6^−/−^* mice, but small mineralization foci were observed, suggesting that a lack of plasma PPi is not the sole mechanism underlying PXE pathology [17]. PPi supplementation inhibits calcification phenotypes in *Abcc6* mutant mice [18,19], but cannot completely eliminate the ectopic calcification phenotypes. Another candidate for PXE is vitamin K. Vitamin K is a cofactor required by gamma-glutamyl carboxylase (GGCX) to convert glutamic acid (Glu) into gamma-carboxylglutamic acid (Gla) [20]. Matrix Gla protein (MGP) undergoes gamma-glutamyl carboxylation and becomes an anti-mineralization factor carboxylated MGP (cMGP) [21,22]. Vitamin K and cMGP were found to be reduced in PXE patients, but *Abcc6^−/−^* mice only displayed decreased cMGP [21,23]. Mutations in GGCX results in cMGP reduction and a PXE-like phenotype [21,24]. Vitamin K was also shown to be effective in reversing its antagonist warfarin-induced calcification in a rat model of arterial calcification [25]. However, vitamin K administration in *Abcc6^−/−^* mice failed to counteract connective-tissue calcification, suggesting that vitamin K may not be the only limiting factor in the pathology in *Abcc6^−/−^* mice [26,27,28]. Hence, the nature of substrates transported by ABCC6 and the pathophysiological mechanisms of PXE remain obscure. There are no effective treatments currently available.

Zebrafish *abcc6a* mutants were previously generated through the ENU mutagenesis and CRISPR/Cas9 technique [29,30,31]. Mutations in zebrafish *abcc6a* result in extensive axial skeleton mineralization [29,31]. Vitamin K treatment reduced ectopic skeleton calcification in the spine of *abcc6a* mutants and knockouts [29]. However, no ectopic fibrosis and calcification phenotypes in PXE-affected tissues such as the eyes and heart have been reported in previously generated zebrafish *abcc6a* mutants, including *grate/abcc6a* missense mutants, *abcc6a* splice site mutants or Cas9-mediated *abcc6a* knockouts [29,31]. *abcc6a* transcripts did not seem to be determined in these *abcc6a* mutants [30]. Thus, the precise effects of zebrafish *abcc6a* mutation on PXE pathology are not well understood.

In this study, we aimed to investigate the hypermineralisation in PXE-affected tissues in *abcc6a* mutants generated by the TALEN technique. We have found that mutations in zebrafish *abcc6a* caused a reduction in *abcc6a* transcripts and protein, resulting in cardiac fibrosis, as well as extensive calcification in the axial skeleton, ocular sclera and Bruch’s membrane. Both PPi and cMGP levels were reduced in the serum and various tissues of *abcc6a* mutants. Interestingly, *abcc6a* mutant hearts displayed the upregulation of ECM family genes (*collagen, fibronectin* and *tenascin*) and key epithelial mesenchymal transition (EMT) genes (*snai1*, *snai2* and *snai3*), suggesting the involvement of *abcc6a* in regulating zebrafish cardiac fibrosis.

## 2. Results

### 2.1. Generation of Zebrafish abcc6a Mutants

**Figure 1:**
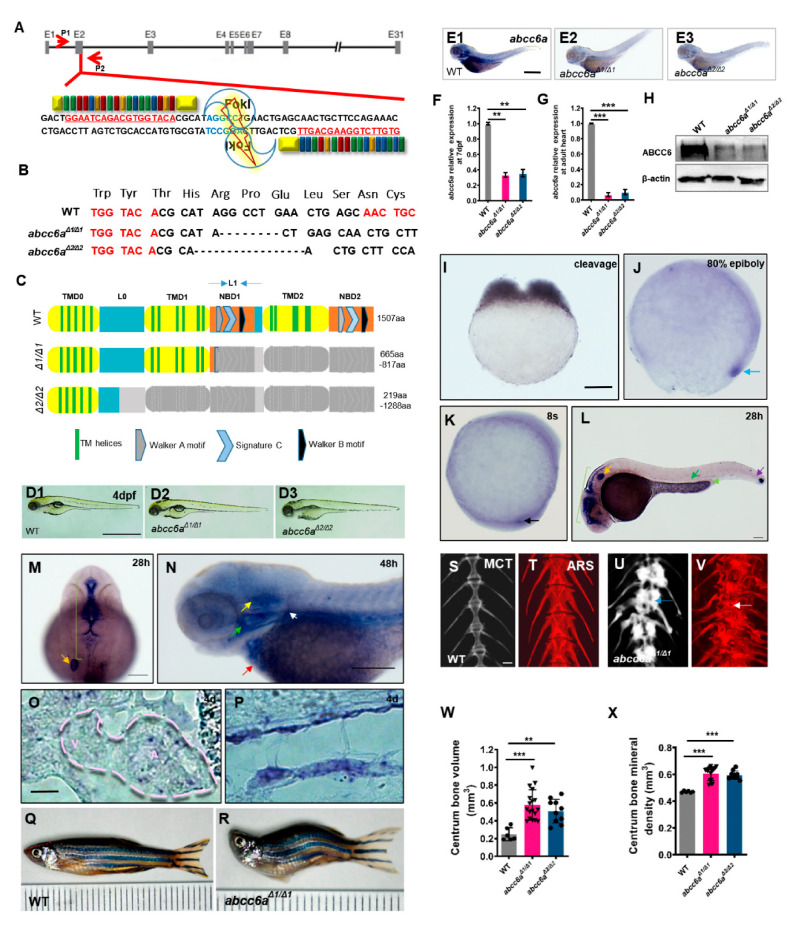
Generation of *abcc6a* mutants and expression patterns in zebrafish. (**A**) Schematic of the zebrafish *abcc6a* gene. Left and right arms of TALEN targeting exon 2 are color-coded to represent 4 repeat-variable diresidues (RVDs) (red, NI = A; green, NN = G; blue, NG = T; orange, HD = C). Nucleotides in red letters and underlined indicate TALEN target sequence. Selected StuI site in the spacer is highlighted with violet. Genomic loci including StuI site is targeted using primer pairs P1 and P2. (**B**) Deletion mutations generated by TALEN at the *abcc6a* gene. The WT sequence is shown at the top. Deletions are indicated by the black dashes. Red letters represent partial TALEN target sequences. Sequences are organized as sets of three bases of in-frame codons. (**C**) Domain structure and predicted amino acid sequence of WT and TALEN mutants. Gray shade indicates regions missing in the mutant proteins. All mutants contain premature stop codon resulting in a lack of most of Abcc6a domains. (**D1**–**D3**) Lateral views showing embryo morphology of *abcc6a^Δ1/Δ1^* (**D2**) and *abcc6a^Δ2/Δ2^* mutants (**D3**) and WT siblings (**D1**) at 4 dpf. (**E1**–**E3**) Whole-mount in situ hybridization (WISH) analysis of *abcc6a* expression at 4 dpf in *abcc6a^Δ1/Δ1^* (**E2**) and *abcc6a^Δ2/Δ2^* mutants (**E3**) and wild-type siblings (**E1**). (**F**,**G**) *abcc6a* expression levels were examined using real-time PCR analysis in *abcc6a^Δ1/Δ1^* and *abcc6a^Δ2/Δ2^* mutant larvae at7 dpf (**F**) and adult hearts (**G**). Expression levels were normalized to the expression of *β-actin*. Data are mean ± SEM from 20 larvae and 6 hearts for each group. ** *p* < 0.01, *** *p* < 0.001, Student’s *t*-test (unpaired, two-tailed). (**H**) Western blot analysis showing ABCC6 protein levels in *abcc6a^Δ1/Δ1^* and *abcc6a^Δ2/Δ2^* mutants and WT larvae at 7 dpf. (**I**–**K**) WISH showing ubiquitous *abcc6a* expression at the two-cell stage (**I**), the 80%-epiboly stage (**J**), and 8 somite stages (**K**). Blue arrow indicates dorsal forerunner cells (**J**) and black arrow indicates Kupffer’s vesicle (**K**). (**L**,**M**) *abcc6a* is detected in the pronephric duct (L; lime arrowhead), aorta-gonad-mesonephros (AGM) region (L; green arrow), midbrain–hindbrain boundary and hindbrain (L, M; green bracket) and otic vesicle (L, M; orange arrow). **(N)** At 48 hpf, *abcc6a* expression is detected in the heart (red arrow), opercula (green arrow), cleithrum (white arrow), and ear (yellow arrow). (**O**,**P**) Lateral views of the *abcc6a* transcripts expression in frozen sections of heart (**O**) and notochord (**P**) at 4 dpf. Pink dashes indicate heart outlines. V, ventricle. A, atrium. (**Q**,**R**) *abcc6a^Δ1/Δ1^* mutants exhibit malformed adult body axis curvature and short body length at 8 months post fertilization (mpf). Dotted red line indicates extent of the skull uplift. Small compartment represents 1 mm. **(S**–**V)** Micro-CT(MCT) scan (**S**,**U**) and Alizarin Red staining (ARS; T, V) of WT and *abcc6a^Δ1/Δ1^* mutants showing vertebral hyperossification. (**W**,**X**) Quantification of the centrum bone volume (**W**) and bone mineral density (**X**) in *abcc6a^Δ1/Δ1^* and *abcc6a^Δ2/Δ2^* mutants. *** *p* < 0.001, Student’s *t*-test (unpaired, two-tailed). Scale bar: 1mm (**D1**–**D3**, **E1**–**E3**); 200 μm (**I**–**K**, O, P, **S**–**V**); 500 μm (**L**,N); 500 μm (**M**).

While several *abcc6a* mutants were previously generated in zebrafish, their mutant phenotypes do not recapitulate the features of human PXE [13,29,30,31]. To investigate the effects of *abcc6a* loss of function in zebrafish, we generated *abcc6a* mutants using an efficient TALEN knockout technology (Figure 1A). Nuclease digestion and sequencing identified the generation of two mutant alleles, named *abcc6a^Δ1/Δ1^* (8bp deletion) and *abcc6a^Δ2/Δ2^* (17bp deletion) (Figure 1B). Both *abcc6a^Δ1/Δ1^* and *abcc6a^Δ2/Δ2^* mutants caused premature termination, leading to truncated proteins with the deletion of nucleotide-binding domains (NBD1 and NBD2), transmembrane domains (TMD0, TMD1, and TMD2), Walker A/B (ATP-binding sites), and signature C (Figure 1C). *abcc6a^Δ1/Δ1^* and *abcc6a^Δ2/Δ2^* mutants were morphologically indistinguishable from wild-type (WT) siblings at four days post fertilization (dpf) (Figure 1(D1–D3)). Unlike the previously reported *abcc6a* mutants [29], *abcc6a^Δ1/Δ1^* and *abcc6a^Δ2/Δ2^* showed the absence of *abcc6a* expression using in situ hybridization analysis (Figure 1(E1–E3)). This might be due to the premature stop codon after the frame shift mutation triggers nonsense-mediated mRNA decay (NMD) [32]. qPCR analyses indicated a significant reduction of *abcc6a* transcripts in *abcc6a^Δ1/Δ1^* and *abcc6a^Δ2/Δ2^* mutant larvae and mutant hearts compared to those measured in WT fish (Figure 1F,G). Furthermore, Western blot analysis determined that Abcc6 protein levels were significantly reduced in both types of mutants at7 dpf, revealing *abcc6a* loss-of-function mutations (Figure 1H). We also found that expression of *abcc6b.1*, *abcc6b.2* and *abcc1* did not increased in *abcc6a^Δ1/Δ1^* and *abcc6a^Δ2/Δ2^* mutants (Figure S1A; data not shown), suggestive of a failure to provoke genetic compensation [33].

We observed that *abcc6a* transcripts were maternally deposited in cleavage-stage embryos (Figure 1I), and then distributed in dorsal forerunner cells throughout blastula embryos (Figure 1J). During somitogenesis, *abcc6a* was expressed in Kupffer’s vesicle (KV) (Figure 1K). At 28 h post fertilization (hpf), *abcc6a* transcripts were enriched in tissues including otic vesicles (OV), the midbrain-hindbrain boundary area, aorta-gonad-mesonephros region, pronephric duct and somite boundary (Figure 1L,M). At 48 hpf and 4 dpf, *abcc6a* was expressed in the heart, opercula, cleithrum, ear and notochord (Figure 1N–P).

At young adult stages, *abcc6a^Δ1/Δ1^* and *abcc6a^Δ2/Δ2^* mutants developed shorter body length and malformed body axis curvature (Figure 1Q,R; Figure S2A,B). About 50% of mutants died at the age of 22 months, compared to WT controls (Figure S1B). Micro-CT scan analyses revealed fusion of the vertebral bodies and intervertebral spaces in adult *abcc6a^Δ1/Δ1^* and *abcc6a^Δ2/Δ2^* mutants compared to WT animals (Figure 1S,U; Figure S2C,E). We performed histopathological staining for calcium and phosphate using Alizarin Red staining (ARS) to detect calcification tissue. We found that adult *abcc6a^Δ1/Δ1^* and *abcc6a^Δ2/Δ2^* mutants suffered from excessive hypermineralisation with calcified nodular lesions in the vertebrae, as compared to adult WT zebrafish (Figure 1T,V; Figure S1C,E; Figure S2D,F). Quantification analyses of centrum bone volume and bone mineral density indicated a marked increase in hypermineralisation in the *abcc6a^Δ1/Δ1^* and *abcc6a^Δ2/Δ2^* mutant vertebrae (Figure 1W,X). Moreover, ocular suborbital and supraorbital bones in *abcc6a^Δ1/Δ1^*mutants became hypermineralized by the ARS assay compared to WT bones (Figure S1D,F). The axial skeleton hypermineralisation phenotypes in *abcc6a^Δ1/Δ1^* mutants generated in this study are consistent with previously reported *grate* missense mutants and CRISPR/Cas9-mediated *abcc6a* knockout mutants [29,31].

### 2.2. abcc6a Mutants Display Ocular Calcification and Fibrosis in Adult Eyes

**Figure 2:**
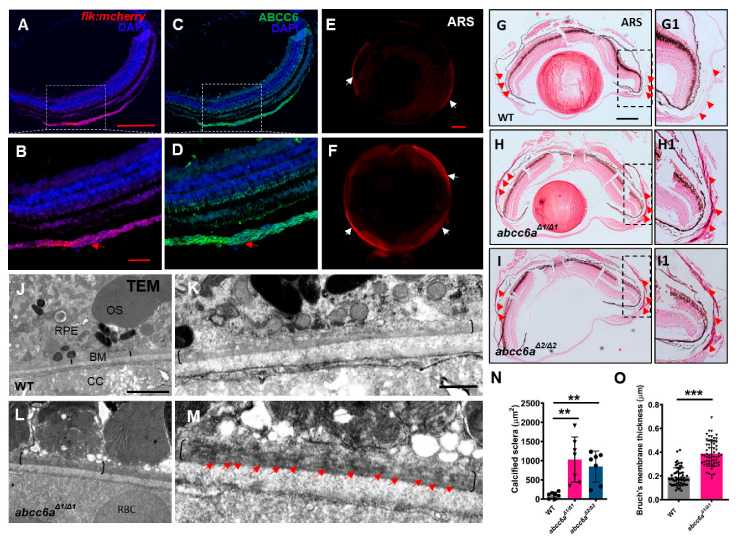
*abcc6a* mutants display ectopic calcification in the eyes. (**A**–**D**) Immunofluorescence results show that Abcc6 is located in the vascular-rich choroidal tissues by co-localization staining (red arrow) of *Tg (flk: mcherry)* fish. (**E**,**F**) Whole mount Alizarin Red staining shows ectopic scleral calcification in isolated eyes of *abcc6a^Δ1/^^Δ1^* mutants. (**G**–**I**; **G1**–**I1**) Alizarin Red staining shows that the scleral layer of the *abcc6a^Δ1/Δ1^* and *abcc6a^Δ2/Δ2^* mutant eyes displays an accumulation of abnormal calcification (**H**,**I**; **H1**,**I1**) compared to WT (**G**,**G1**). (**G1**–**I1**) Higher-magnification images of the dashed boxes in (**G**–**I**). Red arrow marks abnormal calcification of scleral layer in the *abcc6a^Δ1/^^Δ1^* mutant eyes. (**J**–**M**) Transmission electron microscopy showing abnormal thickening and enrichment of dense electron core of Bruch’s membrane in *abcc6a^Δ1/^^Δ1^* mutant eyes (**L**), compared with WT eyes (**J**). (**K**,**M**) Higher-magnification images of the dashed boxes in (**J**,**L**). Black brackets indicate the position of BM, and red arrow indicates position of dense electron core. RPE, retinal pigment epithelium; BM, Bruch’s membrane; CC, choriocapillaris; OS, outer segment; RBC, red blood cell. (**N**) Quantification of area of calcification of sclera of WT (n = 7), *abcc6a^Δ1/^^Δ1^* (n = 7) and *abcc6a^Δ2/Δ2^* fish (n = 7). ** *p* < 0.001, Student’s *t*-test (unpaired, two-tailed). (**O**) Quantification of Bruch’s membrane thickness of WT (n = 6, 10 measuring points per sample) and *abcc6a^Δ1/^^Δ1^* (n = 6, 10 measuring points per sample) fish. *** *p* < 0.001, Student’s *t*-test (unpair, two-tailed). Scale bar: 250 μm (**A**,**C**; **E**,**F**; **G**–**I**); 200 μm (**B**,**D**);62.5 μm (**G1**–**I1**); 2 μm (**J**,**L**); 0.5 μm (**K**,**M**).

Angioid streak in the eye is a typical clinical manifestation in PXE patients [4]. Angioid streak is caused by mineralization and thickening in the Bruch’s membrane, a thin elastin-rich layer behind the pigmented epithelium of the retina [34]. Zebrafish eye divides into sclera, choroid, and retina from outside to inside [35]. The sclera is composed of three tissue layers: episclera containing loose connective tissue; sclera proper, which gives the white color, and the lamina fusca is the innermost zone made up of elastic fibers. To define the Abcc6 location in adult zebrafish eyes, we performed immunostaining analyses using ABCC6 antibody, which was raised in rabbit against human ABCC6 protein. We first validate the ABCC6 antibody specificity by conducting immunofluorescent analyses in human hepatocellular carcinomas cells (HepG2) and mouse renal tubular epithelial cells (IMCD3), in which ABCC6 protein was previously detectable [36,37]. We found that ABCC6 was localized in the plasma membrane in HepG2 and IMCD3 cells (Figure S3A–D), but not negative control cells human retinal pigment epithelium (RPE1) cells and mouse chondrogenic cells (ATDC5) (Figure S3E–H). In zebrafish eyes, we detected Abcc6 in the choroid, a layer of blood vessel and smooth muscle-rich structures (Figure 2A–D; Figure S4A,B). The Bruch’s membrane layer is located between the choroid and retina pigmentation layer [36]. We observed the advanced fibrosis, as assessed by Picrosirius Red staining (PRS) in the *abcc6a^Δ1/^^Δ1^* mutant sclera, in comparison to WT eyes (Figure S4C–F). Notably, ocular sclera in *abcc6a^Δ1/^^Δ1^* mutants became hypermineralized in isolated eyes by whole mount ARS assay (Figure 2E,F). Furthermore, histological analyses revealed extensive calcification in *abcc6a^Δ^^1/^^Δ^^1^* and *abcc6a^Δ2/Δ2^* mutant sclera compared to WT sclera (Figure 2G–I; G1–I1). Quantification revealed that the area of calcification of sclera was significantly increased in *abcc6a^Δ1/^^Δ1^* and *abcc6a^Δ2/Δ2^* mutant eyes (Figure 2N). Consistent with previous studies, transmission electron microscopy (TEM) revealed electron dense material in the mutant Bruch’s membrane (Figure 2J–M), resembling the calcification of Bruch’s membrane in *Abcc6^−/−^* mice and PXE patients [38,39]. Moreover, the Bruch’s membrane thickness was elevated by 63.3% in *abcc6a^Δ1/^^Δ1^* mutants over WT eyes (Figure 2O).

In the intestine, muscularis external and lamina propria contain subepithelial myofibroblasts [40]. In the adult zebrafish intestine, Abcc6 was mostly detectable in muscularis external and lamina propria with partial overlaps with smooth muscle actin (SMA), while some of the enterocytes weakly express Abcc6 (Figure S4G,H). We observed fibrosis accumulation in the thickened muscularis external and lamina propria in *abcc6a^Δ1/^^Δ1^* mutants, compared to that in WT animals (Figure S4I–K). However, calcification in the mutant intestine was not detectable (data not shown). Overall, *abcc6a* mutation caused fibrosis and calcification phenotypes that were similar to pathological PXE symptoms. 

### 2.3. abcc6a Mutation Upregulates Extracellular Matrix Genes and leads to Fibrotic Heart

**Figure 3:**
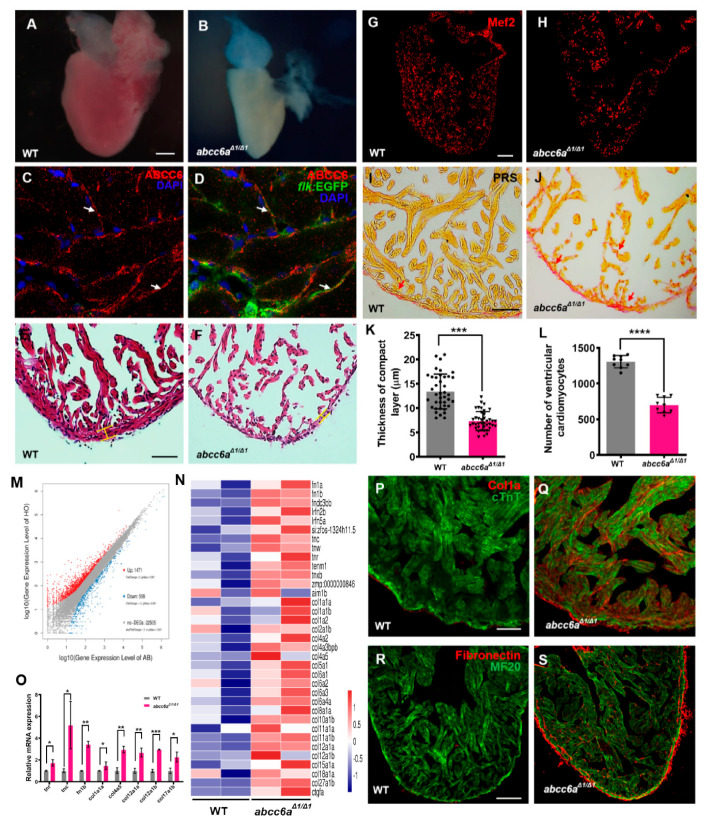
*abcc6a^Δ1/^^Δ1^* mutation upregulates extracellular matrix genes and leads to fibrotic adult hearts. (**A**,**B**) Whole-mounted photographs of adult heart at 8 mpf showing cardiac shrinkage and fibrosis phenotypes in *abcc6a^Δ1/^^Δ1^* (**B**), compared with WT sibling heart (**A**). (**C**,**D**) Abcc6 protein (red) recognized with anti-Abcc6 antibody overlaps with endocardial cells marked by *flk*:EGFP (green) in adult *Tg(flk:EGFP)* heart. Arrows in (**C**,**D**) point to endocardial cells expressing Abcc6. (**E**,**F**) Hematoxylin-eosin (HE) staining exhibits thinner compact layer and fewer myocardial cells in *abcc6a^Δ1/^^Δ1^* heart (**F**), compared to WT sibling hearts (**E**). (**G**,**H**) Representative Picrosirius Red staining of heart sections (yellow for cardiomyocyte fiber, red for collagen) shows superfluous collagen and elastin deposition residing in a compact layer and trabeculae in *abcc6a^Δ1/^^Δ1^* adult hearts (**H**), compared to WT hearts (**G**). (**I**,**J**) Immunofluorescent section images of *abcc6a^Δ1/^^Δ1^* mutant hearts (J) and WT siblings (I), stained with anti-Mef2 antibody. (**K**) Quantitative analysis shows a thinner compact layer thickness in *abcc6a^Δ1/^^Δ1^* adult heart (n = 10, 4 measuring points per sample), compared with WT sibling heart (n = 10, 4 measuring points per sample). *** *p* < 0.001, Student’s *t*-test (unpaired, two-tailed). (**L**) Quantification of the number of cardiomyocyte cells in the same area of WT (n = 9) and *abcc6a^Δ1/^^Δ1^* mutants (n = 9). **** *p* < 0.0001, Student’s *t*-test (unpaired, two-tailed). (**M**) Volcano plots showing differentially expressed genes. 1471 genes are increased in *abcc6a^Δ1/^^Δ1^* mutant hearts and 599 genes expression are reduced, compared to WT hearts. (**N**) Heat map indicates genes upregulated in *abcc6a^Δ1/^^Δ1^* mutant hearts, compared to WT hearts (higher expression in red, lower expression in blue). FC > 1.5, *p* < 0.05. (**O**) qPCR analyses of tenascin family genes (*tnr* and *tnc*) and *fibronectin b* and collagen family genes (*col4a5*, *col12a1a*, *col12a1b* and *col17a1b*) in hearts extracted from *abcc6a^Δ1/^^Δ1^* and *abcc6^+/+^* animals. Data presented as mean ± SEM, n = 3, * *p* < 0.05, ** *p* < 0.01, *** *p* < 0.001, Student’s *t*-test (unpaired, two-tailed). (**P**,**Q**) Immunostaining analyses reveal a robust collagen I in *abcc6a* mutant hearts (**Q**), compared to that in WT hearts (**P**), stained with anti-Col1a (red) and anti-cTnT antibodies (green). (**R**,**S**) Immunostaining analyses show pervading Fibronectin in compact and trabecular layers in *abcc6a^Δ1/^^Δ1^* hearts (**S**) compared to WT hearts (**R**), stained with anti-Fibronectin (red) and anti-MF20 antibodies (green). Scale bar: 100 μm (**A**,**B**; **G**,**H**; **R**,**S**); 50 μm (**E**,**F**); 20 μm (**P**,**Q**).

PXE multisystem disorder progresses with cardiovascular manifestations, including cardiomyopathy, myocardial dysfunction and early myocardial infarction [7]. We investigated whether *abcc6a^Δ1/^^Δ1^* mutations lead to cardiovascular malformation and dysfunction, including cardiac fibrosis and calcification. *abcc6a^Δ1/^^Δ1^* mutant hearts were evaluated at embryonic stages and in adult fish from two to eight months. At approximately eight months of age, *abcc6a^Δ1/^^Δ1^* and *abcc6a^Δ2/Δ2^* mutant heart appeared to be pale and shrunken (Figure 3A,B; Figure S5A,B), suggestive of cardiac fibrosis [41]. However, *abcc6a* mutant hearts appeared to develop normally during embryonic stages (Figure S6A,B) and at two months of age (Figure S6C,D). We found that Abcc6 was detectable throughout the adult zebrafish heart with enrichment in the endocardium marked by *flk:*EGFP (Figure 3C,D). Histological analyses revealed a thinner compact myocardium layer and a smaller trabecular zone in *abcc6a^Δ1/^^Δ1^* and *abcc6a^Δ2/Δ2^* hearts than that in WT sibling hearts (Figure 3E,F,K; Figure S5C,D). Moreover, *abcc6a^Δ1/^^Δ1^* and *abcc6a^Δ2/Δ2^* mutations caused a marked reduction of cardiomyocytes labeled by Mef2, a nuclear cardiomyocyte marker (Figure 3G,H; Figure S5E). Quantification analysis revealed about a 48.0% reduction of ventricular cardiomyocyte number in *abcc6a^Δ1/^^Δ1^* hearts compared to WT sibling hearts (Figure 3L). Importantly, PSR staining revealed significant fibrosis in the compact and trabecular layer in *abcc6a^Δ1/^^Δ1^* ventricles compared to WT cardiac chambers (Figure 3I,J).

To gain insights into the potential mechanism of ABCC6 regulating cardiac fibrosis, we performed RNA sequencing (RNA-seq) using total RNAs extracted from *abcc6a^Δ1/^^Δ1^* mutant hearts and WT sibling hearts at eight months of age. We found that the expression of 1471 genes were increased in *abcc6a^Δ1/^^Δ1^* mutant hearts, compared to WT hearts, whereas the expression of 599 genes were reduced (Figure 3M). Gene Ontology (GO) and heatmap analyses indicated the upregulation of the expression of extracellular matrix (ECM) family genes in *abcc6a^Δ1/^^Δ1^* hearts, such as collagen, fibronectin and tenascin genes in comparison to WT hearts (Figure 3N). Remarkably, the majority of collagen genes, including *col1*, *col2*, *col4*, c*ol5*, *col6*, *col8*, *col10*, *col11*, *col12*, *col15*, *col18*, and *col27* were marked as induced in *abcc6a^Δ1/^^Δ1^* hearts (Figure 3N). Our qPCR assays validated the increased expression of *tnr*, *tnc*, *fn1b*, *col1a1a*, *col4a5*, *col12a*, and *col17a* in *abcc6a^Δ1/^^Δ1^* mutant hearts (Figure 3O). Moreover, immunofluorescent staining analyses revealed an increase in collagen I in *abcc6a^Δ1/^^Δ1^* heart compared to WT heart (Figure 3P,Q; Figure S7A,B). Similarly, *abcc6a^Δ1/^^Δ1^* mutation caused the induction of Fibronectin throughout the mutant ventricle with the enrichment in the myocardial compact layer in comparison to WT ventricles (Figure 3R,S). The upregulation of ECM genes in *abcc6a^Δ1/^^Δ1^* mutant hearts is consistent with their fibrotic phenotypes.

Interestingly, *abcc6a^Δ1/^^Δ1^* mutant hearts displayed the upregulation of key epithelial mesenchymal transition (EMT) key genes (*snai1a*, *snai1b*, *snai2* and *snai3*) and matrix metalloproteinases (MMP) genes (*mmp9* and *mmp13a*) by qPCR analyses (Figure S7C), suggesting the involvement of EMT in Abcc6a-mediated cardiac fibrosis.

### 2.4. Vitamin K Treatment Relieves Ocular Calcification and Cardiac Fibrosis in abcc6a Mutants

**Figure 4:**
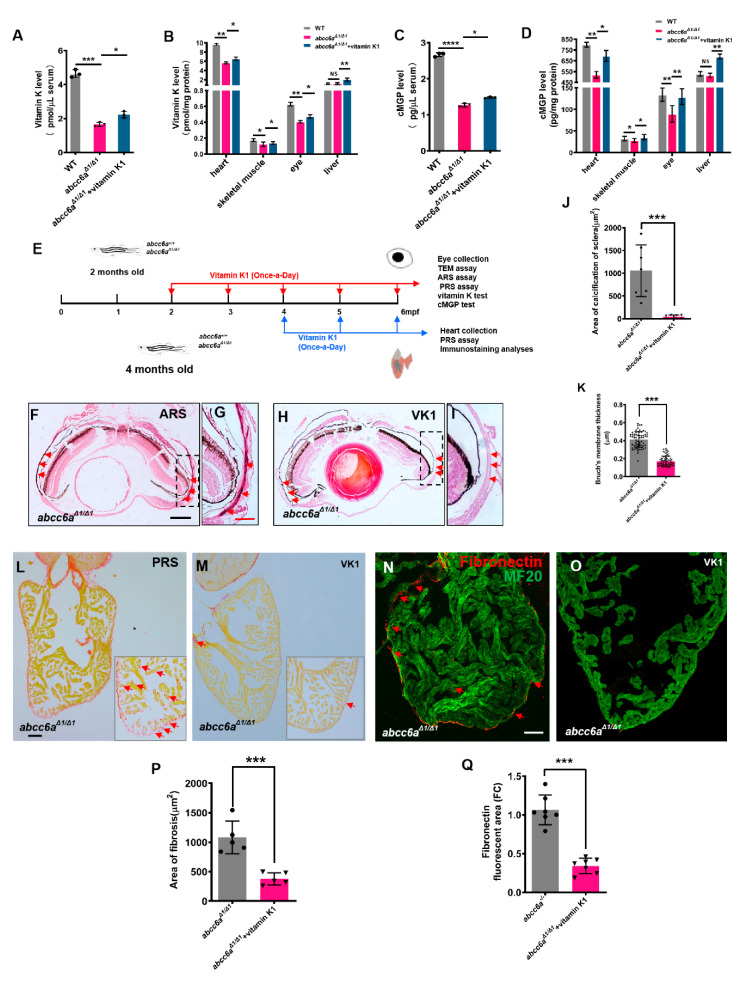
Vitamin K treatment relives ocular calcification and cardiac fibrosis in *abcc6a^Δ1/Δ1^* mutants. (**A**–**D**) Graphical representation of sandwich ELISA measurements for tissue and serum vitamin K and cMGP levels in WT (n = 18) and DMSO/ethanol- (n = 18) or vitamin K1-treated *abcc6a^Δ1^/^Δ1^* fish (n = 18) fish. * *p* < 0.05, ** *p* < 0.01, *** *p* < 0.001, **** *p* < 0.0001, Student’s *t*-test (unpaired, two-tailed). (**E**) Experimental design for once-a-day DMSO/ethanol (0.1%) and vitamin K1 (80 µM) treatment of *abcc6a^+/+^* and *abcc6a^Δ1^/^Δ1^* fish. Red arrows indicate experimental steps, changing water every day for 4 months from 2 months old; for eye collection, TEM, ARS, and PRS assay, vitamin K and cMGP tests. Blue arrows indicate experimental steps for heart collection, PRS assay and immunostaining analyses for 2 months from 4 months of age. (**F**–**I**) Vitamin K rescues abnormal scleral calcification in *abcc6a^Δ1^/^Δ1^* mutant zebrafish. (**G**,**I**) Higher-magnification images of the dashed boxes in (**F**,**H**). Dashed black line indicates approximate plane of resection. Red arrow marks abnormal calcification of scleral layer in the *abcc6a^Δ1^/^Δ1^* mutant eyes. (**J**) Quantification of area of calcification of sclera after 4 months of DMSO/ethanol (n = 7) or vitamin K1 treated treatment in *abcc6a^Δ1^/^Δ1^* fish (n = 7). *** *p* < 0.001, Student’s *t*-test (unpaired, two-tailed). (**K**) Quantification of Bruch’s membrane thickness after 4 months of DMSO/ethanol (n = 13, 8 measuring points per sample) or vitamin K1 treatment in *abcc6a^Δ1^/^Δ1^* fish (n = 15, 8 measuring points per sample). *** *p* < 0.001, Student’s *t*-test (unpaired, two-tailed). (**L**,**M**) Picrosirius Red staining of heart sections shows significantly reduced collagen deposition reside in the compact layer and trabeculae after treatment with vitamin K1 (**M**), as compared to *abcc6a^Δ1^/^Δ1^* mutant hearts after treatment with DMSO/ethanol (L). Red for collagen (L; Red arrow) and yellow for cardiomyocyte fiber. (**N**,**O**) Immunofluorescent section images of adult ventricles stained with anti-Fibronectin and anti-MF20 antibodies from DMSO/ethanol-treated (N) or vitamin K1 treated *abcc6a^Δ1^/^Δ1^* mutant fish (O), showing that vitamin K reduces Fibronectin. (**P**) Quantification of area of fibrosis after 2 months of DMSO/ethanol (n = 5) or vitamin K1 treatment in *abcc6a^Δ1^/^Δ1^* fish (n = 5). *** *p* < 0.001, Student’s *t*-test (unpaired, two-tailed). (**Q**) Quantification of Fibronectin fluorescent area after 2 months of DMSO/ethanol (n = 7) or vitamin K1 treatment in *abcc6a^Δ1^/^Δ1^* fish (n = 7). *** *p* < 0.001, Student’s *t*-test (unpaired, two-tailed). Scale bar: 250 μm (**F**,**H)**; 50 μm (**L**–**O**); 125 μm (**G**,**I**).

Vitamin K plays a role in the regulation of mineralization processes as a co-factor of GGCX in the carboxylation of calcification inhibitors such as MGP [42]. Previous studies reported that PXE patients have low serum levels of vitamin K, but vitamin K treatment was not effective in *Abcc6* knockout mice [26,27,28]. In zebrafish, ectopic mineralization in the vertebrae of in *grate* mutants is partially reversed by vitamin K at the larval stages [29]. Similarly, vitamin K-treated embryos (Figure S8A) showed partially rescued craniofacial (Figure S8B,D) and vertebral hypermineralisation in TALEN-mediated *abcc6a^Δ1/^^Δ1^* mutants (Figure S8C,E). We reasoned that vitamin K and cMGP levels in the serum and tissues might be reduced in zebrafish *abcc6a^Δ1/^^Δ1^* mutants. We utilized ELISA methods to determine endogenous vitamin K levels in the serum and various tissues. We found a reduction of vitamin K levels in the serum, heart, eye, and skeletal muscle in *abcc6a^Δ1/^^Δ1^* mutants in comparison to WT sibling tissues (Figure 4A,B). In contrast, vitamin K levels in the liver were apparently not altered (Figure 4B). This might be due to the absence of *Abcc6a* expression in zebrafish liver. Alternatively, vitamin K is preferentially transported to the liver for the activation of coagulant factors and other metabolisms at the expense of extrahepatic vitamin K requirement, if vitamin K is insufficient [43]. Accordingly, cMGP levels in the serum (Figure 4C) and diverse organs, including the heart, eye, and skeletal muscle, were reduced in *abcc6a^Δ1/^^Δ1^* mutants (Figure 4D), suggesting that hypermineralisation in *abcc6a^Δ1/^^Δ1^* mutants is due to low cMGP levels. Similarly, we found that cMGP levels had no significant changes in *abcc6a^Δ1/^^Δ1^* mutant livers (Figure 4D). Recent studies have reported that pyrophosphate (PPi) is an anti-mineralization factor, and reduced PPi concentrations were evident in PXE patients and *Abcc6^−/−^* mice [15]. We measured PPi levels using pyrophosphate assay, and found a significant reduction in the serum, heart and eye in *abcc6a* mutants compared to WT siblings (Figure S9A,B), suggestive of a conserved role of Abcc6 in regulating PPi levels in zebrafish.

To assess whether vitamin K administration can reverse ocular calcification deposits in *abcc6a* mutants, we administered 80 μM vitamin K1 to 2-month-old adult *abcc6a^Δ1/^^Δ1^* fish for 120 days (Figure 4E). Vitamin K concentrations in the serum and various tissues, were increased in the vitamin K-treated mutant zebrafish (Figure 4A–B). We found that vitamin K treatment diminished calcification deposits in the ocular sclera in *abcc6a^Δ^^1/^^Δ^^1^* mutant eyes, compared to vehicle-treated mutants (Figure 4F–J). Furthermore, the thickening of Bruch’s membrane, an important clinical PXE phenotypes, was also partially rescued by vitamin K administration (Figure 4K).

Next, we assessed the effects of vitamin K on cardiac fibrosis by administering 80 μM vitamin K1 to 4-month-old *abcc6a^Δ1/^^Δ1^* mutant zebrafish for 60 days (Figure 4E). PRS analyses indicated that overall fibrosis in vitamin K-treated ventricles was reduced compared to vehicle-treated mutant hearts (Figure 4L,M,P). Furthermore, vitamin K administration largely diminished Fibronectin, a component in ECM, in *abcc6a^Δ1/^^Δ1^* mutant hearts (Figure 4N,O,Q). As expected, cMGP levels in the serum and various tissues, including eyes and heart, were increased (Figure 4C,D).

## 3. Discussion

PXE is a pleiotropic, multisystem disorder with considerable heterogeneity [11]. Zebrafish models have proven to be useful for a broad range of diseases. Previously generated *abcc6a* zebrafish mutants, including the *grate* missense mutants, *abcc6a* splice site mutants and Cas9-mediated *abcc6a* knockouts, develop the similar hypermineralisation in the axial skeleton [29,30,31]. However, no calcification phenotypes have been identified in PXE-affected tissues in these zebrafish mutants, since the axial spine calcification was not observed in PXE patients and *Abcc6^−/−^* mice.

The mineralization phenotype in the axial skeleton was consistent in TALEN-induced *abcc6a* mutants generated in this study. In addition, *abcc6a* mutants induced by TALEN display extensive ocular calcification and increased cardiac fibrosis, which failed to be found in previous zebrafish mutants [29,30,31]. This might be due to the possibility that *abcc6a* transcripts were not determined, and heart and eyes were not carefully examined in previous zebrafish *abcc6a* mutants [30]. Notably, ectopic dense core is prominent in the Bruch’s membrane in TALEN-mediated *abcc6a^Δ1/^^Δ1^* mutants by TEM analyses, consistent with previous studies on *Abcc6* knockout mice and PXE patients [38,39]. Zebrafish *abcc6a* mutants at the age of six months developed the progression of cardiac fibrosis with increased ECM genes and a loss of cardiomyocytes. This might explain the progressive lethality of *abcc6a* mutants at the age of 22 months. In agreement with our findings, cardiac muscle in Cas9-mediated zebrafish *abcc6a^Δ1/^^Δ1^* mutants appears to be sparse compared to WT hearts from histological analyses [29]. It is noteworthy that myocardial fibrosis and cardiomyocyte loss have not been reported as phenotypes associated with either human PXE or *Abcc6* knockout mouse models [39,44]. Arterial blood vessel calcification in the heart is evident in PXE patients and *Abcc6* knockout mice on a C57BL/6J (B6) background [45,46]. However, dystrophic cardiac calcinosis (DCC) caused by *Abcc6* mutations in murine inbred strains C3H/HeJ and DBA/2J developed a progressive myocardial fibrosis and calcification [47,48], suggesting that different murine genetic backgrounds give rise to distinct cardiac phenotypes. These findings implicate the combination of cardiac vascular calcification with increased myocardial fibrosis, which might underly the PXE cardiovascular pathology. It will be important to assess the expression of *fibronectin* and *collagen I* in murine *Abcc6* knockout hearts. Recent studies report that ABCC6 transporter facilitates hepatic release of ATP into the circulation to produce a potent mineralization inhibitor PPi [16]. PXE patients and *Abcc6^−/−^* mice displayed reduced PPi levels in the serum [15]. We have also found that PPi levels in the serum and tissues were decreased in zebrafish *abcc6a* mutants, suggesting a conserved role of Abcc6 in regulating PPi homeostasis. Future studies are required to test whether PPi supplementation can reverse ectopic mineralization in zebrafish *abcc6a* mutants.

Zebrafish is a vertebrate model with a complement of genes that is similar to that in mammals. While zebrafish A*bcc6a* is considered as an orthologue of human *ABCC6*, Abcc6a is only 47% identical at the amino-acid level to the human ABCC6, whereas murine Abcc6 is 79% identical to human ABCC6 [49]. We and others observed that Zebrafish *abcc6a* was expressed in the cleavage-stage embryos, and enriched in the developing bone, eye and heart, but not the liver (unlike mammalian ABCC6/Abcc6). Furthermore, in zebrafish *abcc6a* mutants, ectopic calcification was only detectable in the axial skeleton and eyes, recapitulating a small part of PXE manifestations. In contrast, *Abcc6* knockout mice faithfully mirror the human PXE symptoms, including extensive calcification in the skin, eyes and arterial blood vessels [30,46,50]. Although vitamin K treatment of zebrafish *abcc6a* mutants partially reversed the vertebral [29] and ocular calcification and cardiac fibrosis, vitamin K supplement in *Abcc6^−/−^* mice could not prevent or reverse PXE-related mineralization in calcified tissues [26,27,28]. These differences might be due to distinct genomes and pathophysiologies in teleost zebrafish and mammals. For example, zebrafish genome is partially duplicated, and *abcc6a* and *abcc6b* are duplicated genes located on chromosomes 3 and 6 in the genome, respectively [49]. Future studies will be required to construct *abcc6b* single mutants and generate *abcc6a*/*abcc6b* double mutants to examine the mineralization processes in these single and double mutants. Nevertheless, zebrafish serve as a vertebrate model to study Abcc6 functions and its action mechanisms, shedding light on the divergence of Abcc6-mediated mineralization processes in vertebrates and mammals. We believe that the combination of animal model systems with human PXE genetics and cell biology will discover natural substrates transported by ABCC6, as well as additional mechanisms in preventing aberrant mineralization of connective tissues, which will be relevant to develop effective therapeutic interventions in PXE medicine.

## 4. Materials and Methods

### 4.1. Zebrafish Maintenance

All zebrafish used for the experiments, including wild type (AB), mutants, and transgenic lines, were maintained according to standard protocols. The transgenetic lines in this study include *Tg(flk:EGFP)* and *Tg(flk:mcherry*). Approval of experiments, as well as advise on animal care and research were received from the East China Normal University Animal Care committee (Approval ID: bmy20190401, 1 July 2019)

### 4.2. TALEN Design and Microinjection

Two plasmids of TALENs targeting exon 2 of zebrafish *abcc6a* (ENSDARG00000016750) were constructed using the FastTALE^TM^ TALEN Assembly Kit from SiDanSai Biotechnology. The left side TALEN sequence was designed as 5′-GGAATCAGACGTGGTACA-3′, and the right side sequence as 5′-GTGTTCTGGAAGCAGTT-3′. Individual TALE repeats containing repeat-variable diresidues (RVDs) recognize a single nucleotide using simple rules (TALE code: NG = T, HD = C, NI = A, NN = G) [42]. TALEN mRNAs were synthesized by in vitro transcription using the SP6 mMESSAGE mMACHINE Kit (Thermo Fisher Scientific, Carlsbad, CA, USA). Different doses of mRNA (50–200pg) encoding each of two TALEN arms were injected into the cytoplasm of one cell–stage zebrafish embryos. The genomic DNA was extracted from a mixture of five embryos at 24hpf in accordance with the manufacturer’s manual for the genome extraction kit (Takara). Fragments containing the TALEN target site were amplified by PCR (TransGen Biotech) and digested with StuI to examine the efficiency of TALENs. The genetic selection method was used to obtain the stable *abcc6a* mutants.

### 4.3. In Situ Hybridization

In situ hybridization in zebrafish whole-mount embryos and heart cryosections was performed, as previously described [51]. PCR fragments of *abcc6a* and *cmlc2* amplified from zebrafish cDNA were subcloned into pGEM-T vector (Promega). Embryos and heart cryosections were imaged on a Leica M205 FA dissecting scope equipped with Nikon Digital Sight DS-Ri1 camera (Nikon,Tokyo, Japan).

### 4.4. Real-Time RT-PCR

Total RNAs were separately extracted from 3 groups of 30 embryos or 6 adult hearts using TRIzol reagent (Invitrogen) and reverse transcribed using PrimeScript^™^ II 1st Strand cDNA Synthesis Kit (Takara, Shiga, Japan). The experiment was operated in a 384-well plate on biological replicates with SYBR Premix Ex Taq II (Takara) using a Roche LightCycler 480 II system. The primers used in real-time PCR are listed in Supplementary Table S1.

### 4.5. Immunofluorescence Analysis and Measurement for the Cardiomyocyte Number

Whole-mount hearts were extracted, fixed and cryosectioned. All adult heart cryosections had 10 µm thickness. The following primary antibodies were used in this study: anti-Mef2 (Santa Cruz, Dallas, TX, USA); anti-Abcc6 (Santa Cruz); anti-cTnT (DSHB, Iowa City, IA, USA); anti-MF20 (MHC; DSHB); anti-Collagen Ia (Abcam, Cambridge, CB2 0AX, UK); anti-Fibronectin (Sigma-Aldrich, Saint Louis, MO, USA). Secondary antibodies included Alexa Fluor 488 goat against mouse IgG (H + L) (1:1000; Life Technologies, A11001;Thermo Fisher Scientific, Carlsbad, CA, USA), Alexa Fluor 594 goat against mouse IgG (H + L) (1:1000; Life Technologies, A11005), Alexa Fluor 594 goat against rabbit IgG (H + L) (1:1000; Life Technologies, A11012) and Alexa Fluor 488 goat anti-rabbit (1:1,000; Life Technologies, A11008). Confocal images were obtained using a Zeiss LSM710 laser scanning confocal microscope.

To quantify the cardiomyocyte number, one section showing the largest area was selected from each heart, and images were taken using a 10× objective. The number of Mef2+ was manually counted using Image J software within the same region of the ventricle. WT and mutant hearts were selected for statistical count. Nine cardiac cryosections were taken from WT and mutant hearts for statistical count.

### 4.6. RNA-seq Analysis

Adult hearts were pooled and rapidly frozen in an ultra-low-temperature refrigerator for short preservation. Total RNAs were extracted and performed by Wuhan BGI Company. WT and *abcc6a^Δ1/^^Δ1^* mutant samples were tested on the BGIS EQ-500 platform. The average mapping ratio with reference genome was 90.44% and the average mapping ratio with gene was 72.88%. The average output of each sample was 21.84 M and a total of 24,575 genes were detected. Bowtie2 was used to compare clean reads to reference sequences, and RSEM was used to calculate the expression levels of genes and transcripts. Gene Ontology (GO) and Kyoto Encyclopedia of Genes and Genomes (KEGG) classification and functional enrichment were performed for differential gene detection. The FPKM values per gene for AB and HO samples are listed in Supplementary Table S2.

### 4.7. Paraffin Section and Immunochemical Staining

The fresh tissues were extracted and fixed with 4% paraformaldehyde at 4 °C for 24 h. Fixed tissues were dehydrated, embedded in paraffin and sectioned at 5 µm thickness. Paraffin sections were dewaxed in xylene solution, treated in gradient ethanol and soaked in distilled water. The slides were dipped in hematoxylin-eosin dye kit (G1005; Servicebio, Wuhan, China), Masson dye kit (Servicebio, G1006) and Picrosirius staining (Servicebio, G1018) and rinsed. Finally, well-stained films were dried, sealed, and examined by microscope.

### 4.8. Micro Computed Tomography (Micro-CT)

Six or eight-month-old zebrafish were anesthetized with 0.03% tricaine (Sigma, USA) and gently wrapped in parafilm (Sangon Biotech, Shanghai, China). The whole body was scanned in a SkyScan 1176 high-resolution micro-CT imaging system (SkyScan Bruker, Karlsruhe, BW, Germany). Equipment operating parameters were set as follows: spatial resolution of 9 μm pixel size; X-ray tube voltage of 40 kV; X-ray tube current of 595 μA; no filter and 0.7° rotation step over 180°. Images were reconstructed using SkyScan CT Analyser software (Version: 1.15.2.2), as described in Reference [52]. The region of interest was obtained using CTvox and CTAn 3D analysis software, including the anterior craniofacial part and body axis. All parameters were obtained by means of the “Double Time Cubes” 3D reconstruction method.

### 4.9. Skeletal Staining

Larvae were anesthetized with 0.03% tricaine (Sigma-Aldrich) and fixed in 4% paraformaldehyde (PFA) in phosphate buffered saline (PBS). The protocol for two-color acid-free bone and cartilage staining was adapted from the method previously described in [53]. For adult fish staining, samples were fixed for 1–7 days according to the sample stages in 3.5% paraformaldehyde at 4 °C and incubated in acetone to remove fat for 24–48 h. Next, tissues were washed with PBS with 0.1% Tween-20 twice and incubated in a saturated aqueous solution of sodium tetraborate for 1 day. Then tissues were digested with 10 mg/mL trypsin in 60% sodium tetraborate overnight. Bones were stained with 0.04mg/mL Alizarin red S (Sigma-Aldrich, Saint Louis, MO, USA) in 1% KOH for 1–7 days, followed by washing in 0.1% Tween in 1×PBS. The skin and scales were carefully removed manually. The skeleton was macroscopically imaged on a Leica M205 FA dissecting scope and elaborated by a Zeiss LSM710 laser scanning confocal microscope.

### 4.10. Vitamin K Administration

Vitamin K1 (Sigma-Aldrich) was dissolved in 1:1 DMSO/ethanol to a stock concentration of 40 mM and diluted in aquarium water to 80 µM for bath treatment. To assess whether vitamin K administration could reverse ocular calcification deposits in *abcc6a^Δ1/^^Δ1^* mutants, 2-month-old adult *abcc6a^Δ1/^^Δ1^* fish were treated with 80 μM vitamin K1 or 1% DMSO/ethanol for 120 days. To assess the effects of vitamin K on cardiac fibrosis 4-month-old *abcc6a^Δ1/^^Δ1^* mutant zebrafish were administered 80 μM vitamin K or 1% DMSO/ethanol for 60 days. Animal density was maintained at 10–12 fish/L.

To detect the effect of vitamin K on the accumulation of excessive calcification in embryonic mutants, we collected embryos by the mating of heterozygous parents, developed in clean plastic dishes. Then we added 80 μM vitamin K1 or 1% DMSO/ethanol from 24 h, changed the water every day, and continued to collect embryos after nine days. The phenotypes of embryos were determined by tail cutting and PCR identification.

### 4.11. Serum and Tissue Collection

For serum collection, the groups of WT fish and *abcc6a^Δ1/^^Δ1^* mutant fish treated with DMSO/ethanol and vitamin K1 for 120 days were chosen. After removing the eyeballs of zebrafish, we immediately used a 10 μL micro syringe to draw an equal amount of fresh blood into an Eppendorf tube and centrifuged it at 2–8 °C 1000× *g* for 15 min, and the supernatant was directly detected. The serum of each group of samples required three fish, and the experiment was repeated three times. Tissue collection and serum collection of fish occurred at the same time. Hearts, eyes, skeletal muscle, livers and other tissues of zebrafish were removed to pre-cooled PBS (containing protease inhibitor). After washing and breaking the tissue liquid with a needle, they were transferred to a tissue homogenizer, fully ground on ice, and left for 30 min. After repeating, the homogenate was centrifuged at 5000× *g* for 10 min, and the supernatant was taken. BCA protein was determined and the same protein concentration was calibrated for subsequent detection. Each group contained three fish, and the experiment was repeated three times.

### 4.12. ELISA Assay

This kit used the double antibody sandwich method to determine the level of vitamin K1 (VK1) in fish (Shanghai Hengyuan Biological Technology Co., Ltd., H-42702, Shanghai, China). The monoclonal antibody vitamin K1 was added into the micropore and combined with horseradish peroxidase (HRP) to form a complex. After polyester, tetramethylbenzidine (TMB) was added to develop color. Under TMB catalysis, it was transformed to blue. There was a positive correlation between the color and the fish vitamin K1 in the fish, measured at 450 nm by enzyme-linked immunosorbent assay. The absorbance (OD value) of fish vitamin K1 in the sample was calculated by the standard curve. To determine the cMGP levels, a human matrix gamma carboxyglutamic acid protein (cMGP) detection kit was selected (Shanghai Hengyuan Biological Technology Co., Ltd., H-11621, Shanghai, China). The purified HRP labeled cMGP antibody was coated on the microporous plate to form a complex. After thorough washing, the substrate TMB was added for color rendering. The experiment was repeated three times.

### 4.13. Pyrophosphate Assay

The Pyrophosphate Assay Kit (ab234040) provides a fast and convenient method to determine the free PPi levels in serum, heart and eye in adult zebrafish. PPi produced during biotic processes is detected through a series of reactions which utilize a PPi enzyme mix and PPi probe, generating a stable product that can be quantified by colorimetric readout. Generated color (OD 570 nm) intensities are directly proportional to the concentrations of PPi, enabling precise measurements.

### 4.14. Transmission Electron Microscopy

The eyeballs of zebrafish were taken out within 1–3 min, and fixed in the electron microscope fixative for about half an hour. After the tissue became hard, it was trimmed with a blade to 2 mm × 2 mm. After the discarded part was removed, the tissue was immediately put into the electron microscope fixed solution for 2 h at room temperature, and then transferred to 4 °C for preservation. Tissues avoiding light were fixed with 1% OsO4 in 0.1 M PBS (pH 7.4) for 2 h at room temperature. After removing the OsO4, the tissues were rinsed in 0.1 M PBS (pH 7.4) 3 times for 15 min each. Different concentrations of alcohol solution were added to the tissues in order to dehydrate it for 20 min each time, and 100% acetone twice for 15 min each time. The tissues were put in the 1:1 acetone/812 embedding agent at 37 °C for 2–4 h, 1:2 acetone/812 embedding agent at 37 °C infiltration overnight and pure 812 embedding agent at 37 °C for 5–8 h. The pure 812 embedding agent was poured into the embedding plate, and the sample was inserted into the embedding plate, and then placed in the oven at 37 °C overnight. The embedding board was placed in an oven at 60 °C for 48 h and the resin block was taken out for use. The resin block was ultra-thin sectioned with a 60–80 nm ultra-thin microtome with 150-mesh square Chinese film copper mesh. The copper mesh was stained in a 2% uranyl acetate saturated alcohol solution for 8 min in the dark, washed with 70% alcohol 3 times and ultrapure water 3 times. Then 2.6% lead citrate solution was stained with carbon dioxide for 8 min and washed with ultrapure water for 3 s. The copper mesh slices were placed in a copper mesh box and dried overnight at room temperature. TEM photographs (H-7700) were taken by the ECNU Multifunctional Platform for Innovation (004).

### 4.15. Statistical Methods

Statistical analysis was performed with GraphPad Prism 5 Project (GraphPad Software Inc, San Diego, CA, USA). Differences between the parameters in the two groups were detected using Student’s t-test. All experiments were performed in more than triplicate. *p* < 0.05 was regarded as statistically significant. * *p* < 0.05, ** *p* < 0.01, *** *p* < 0.001, **** *p* < 0.001.

## Data Availability

The data presented in this study are available in supplementary material.

## Supplementary Materials

Supplementary Materials can be found at https://www.mdpi.com/1422-0067/22/1/278/s1.
