# Supplementary material for: Disruption of Abcc6 Transporter in Zebrafish Causes Ocular Calcification and Cardiac Fibrosis
Source: Int J Mol Sci. 2020 Dec 29;22(1):278. doi: 10.3390/ijms22010278 (PMC7795442; doi:10.3390/ijms22010278)
Supplement: Supplementary file 1 [file ijms-22-00278-s001.zip › ijms-1002133-re-check-supplementary/Supplemental Figures sjj _1219.pdf]

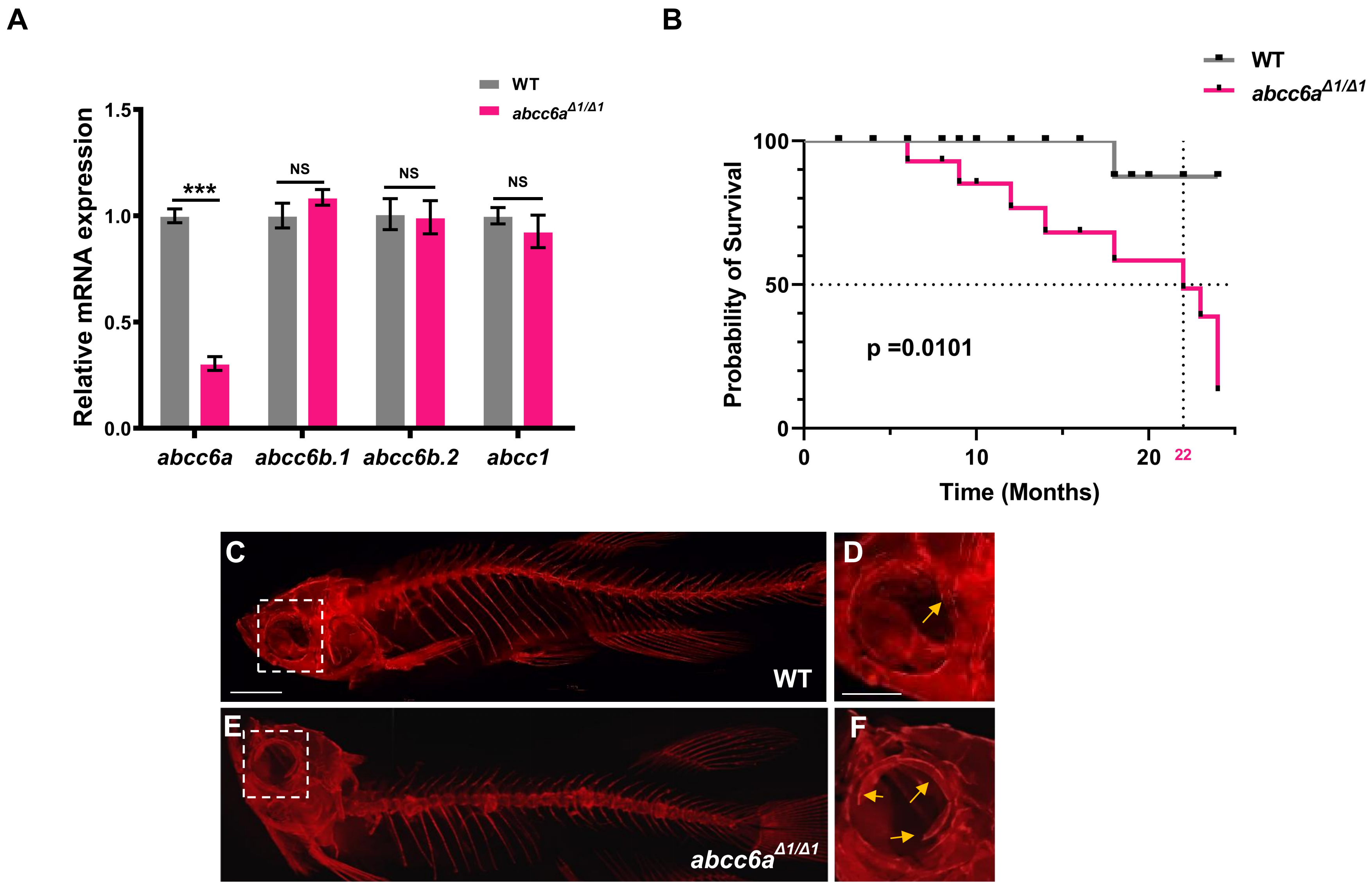


**Fig. S1 Kaplan-Meier survival curves of *abcc6a* mutation**

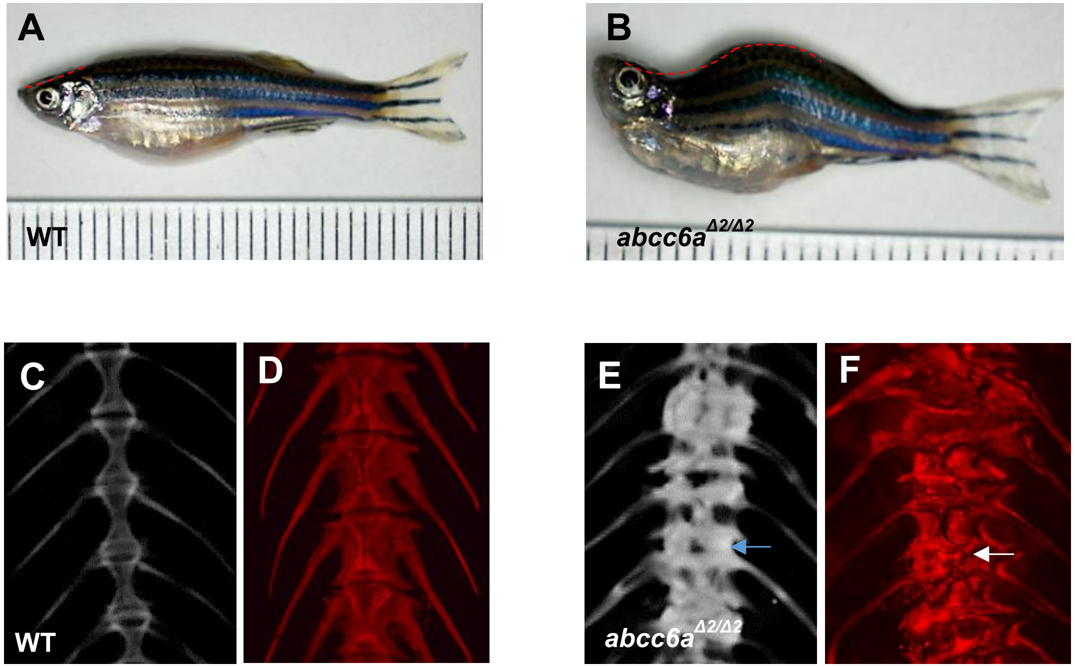


**Fig. S2** ***abcc6a*$^{\Delta2/\Delta2}$ mutants display vertebral hypermineralisation**

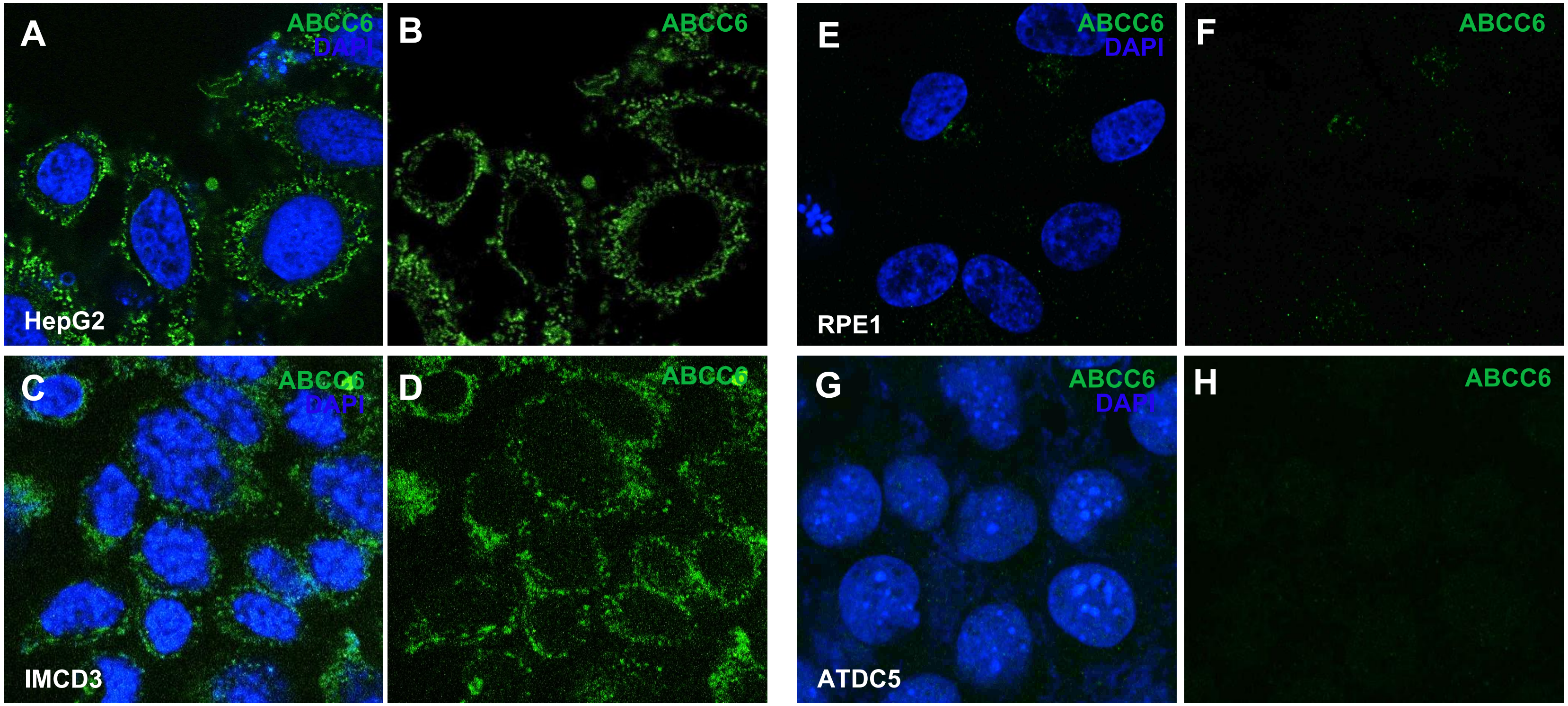


**Fig. S3  Validation of  ABCC6 antibody in several human and mice cell lines**

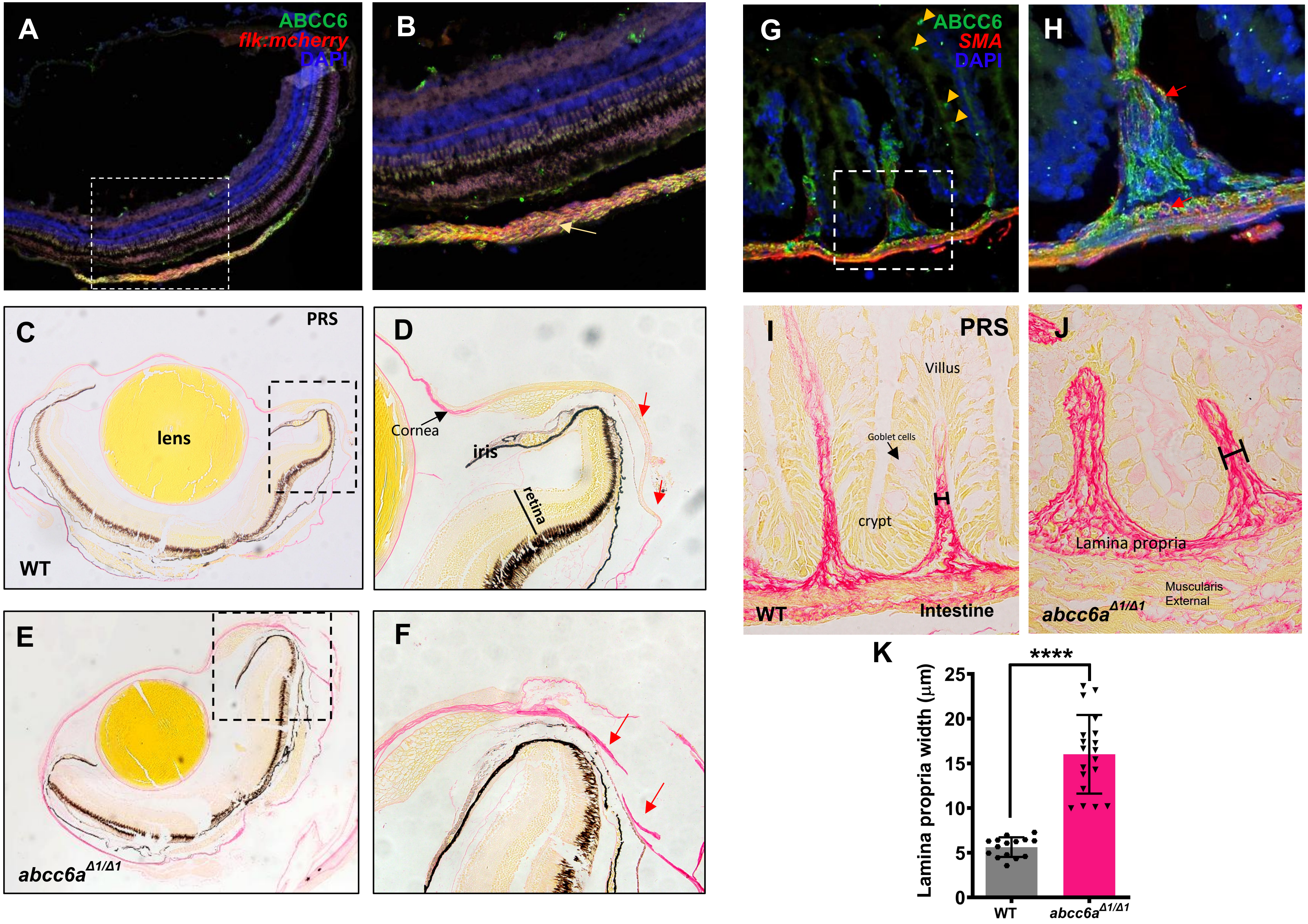


# Fig. S4 *abcc6a* mutants display ocular fibrosis in adult eyes

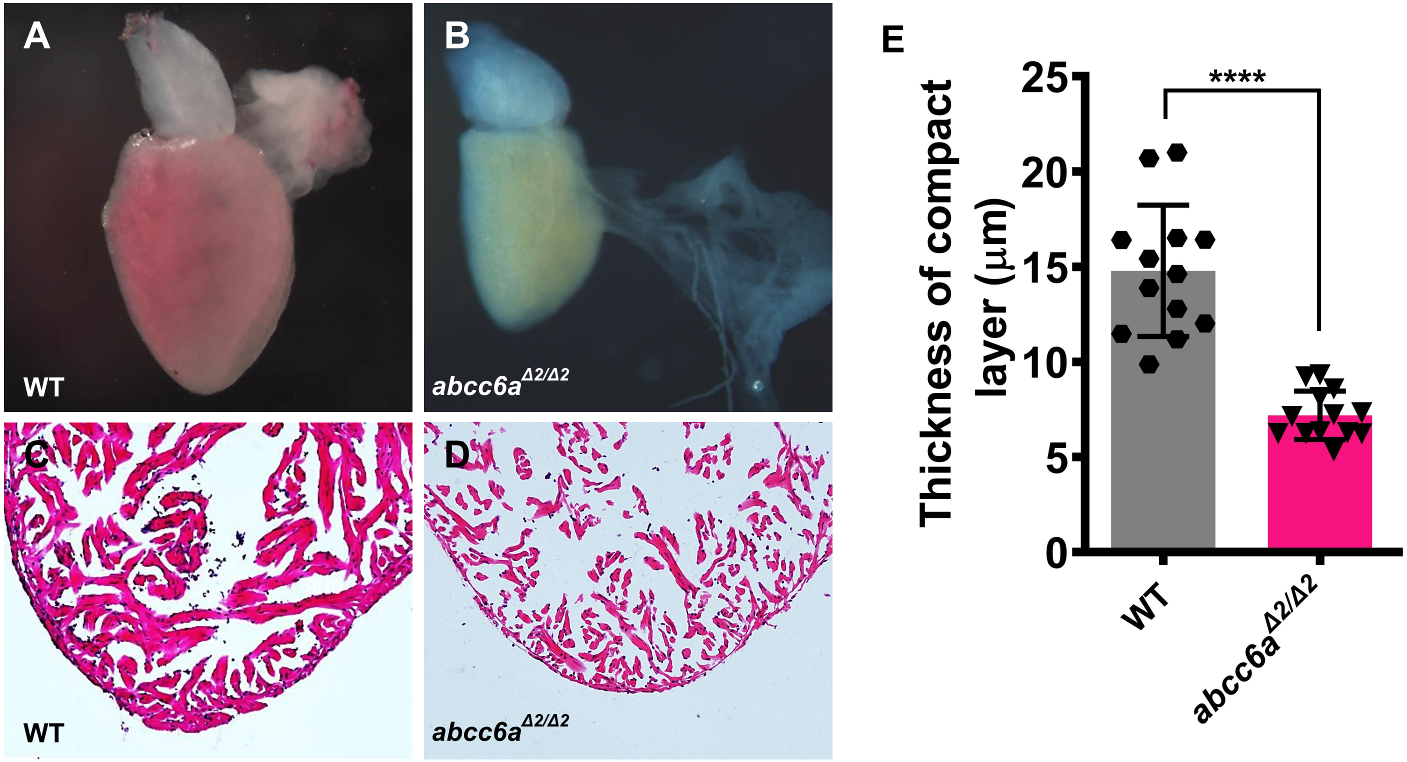


**Fig. S5** ***abcc6a***$^{\Delta 2/\Delta 2}$ **mutants show serious heart malformations**

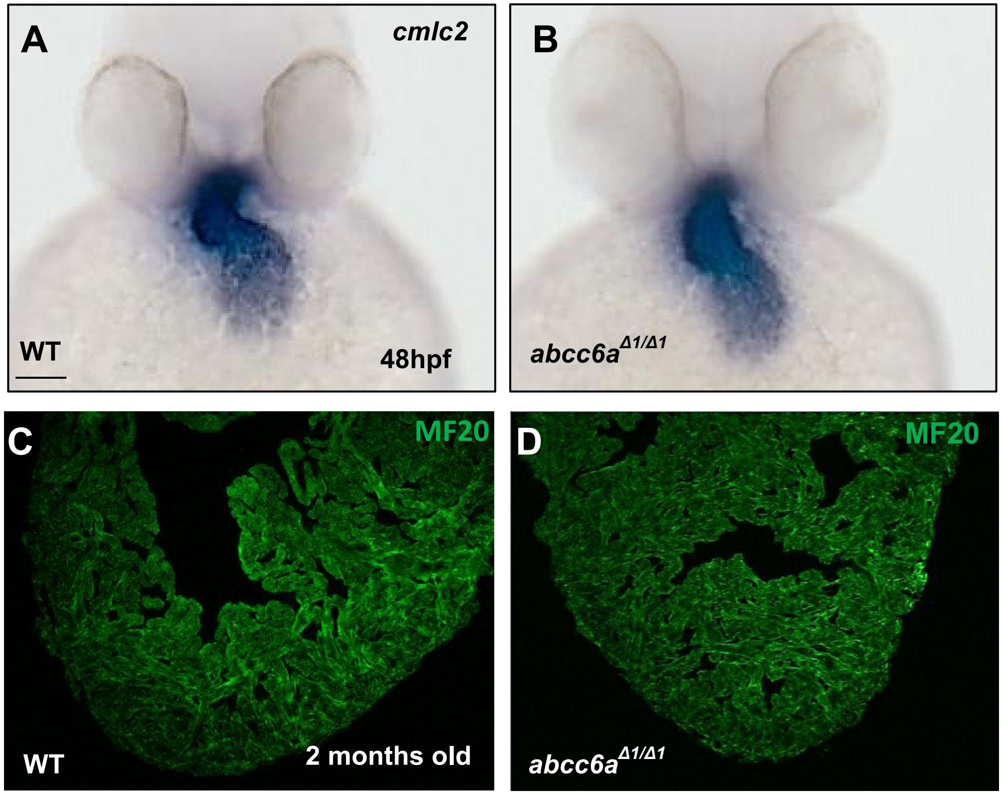


**Fig. S6 *abcc6a* mutant hearts develop normally during embryonic and young adult stages**

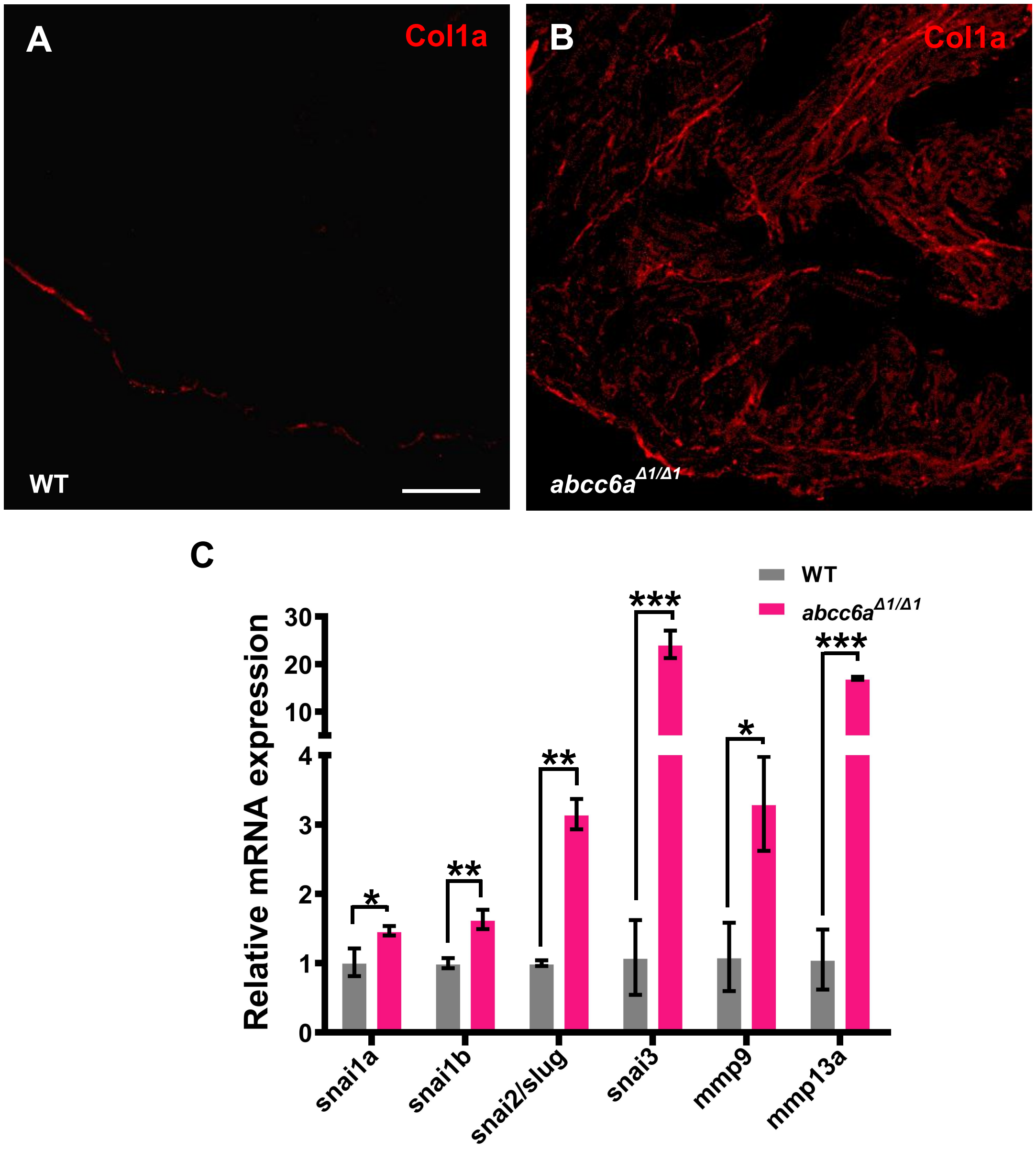


**Fig. S7** ***abcc6a*** **deficiency upregulates EMT genes**

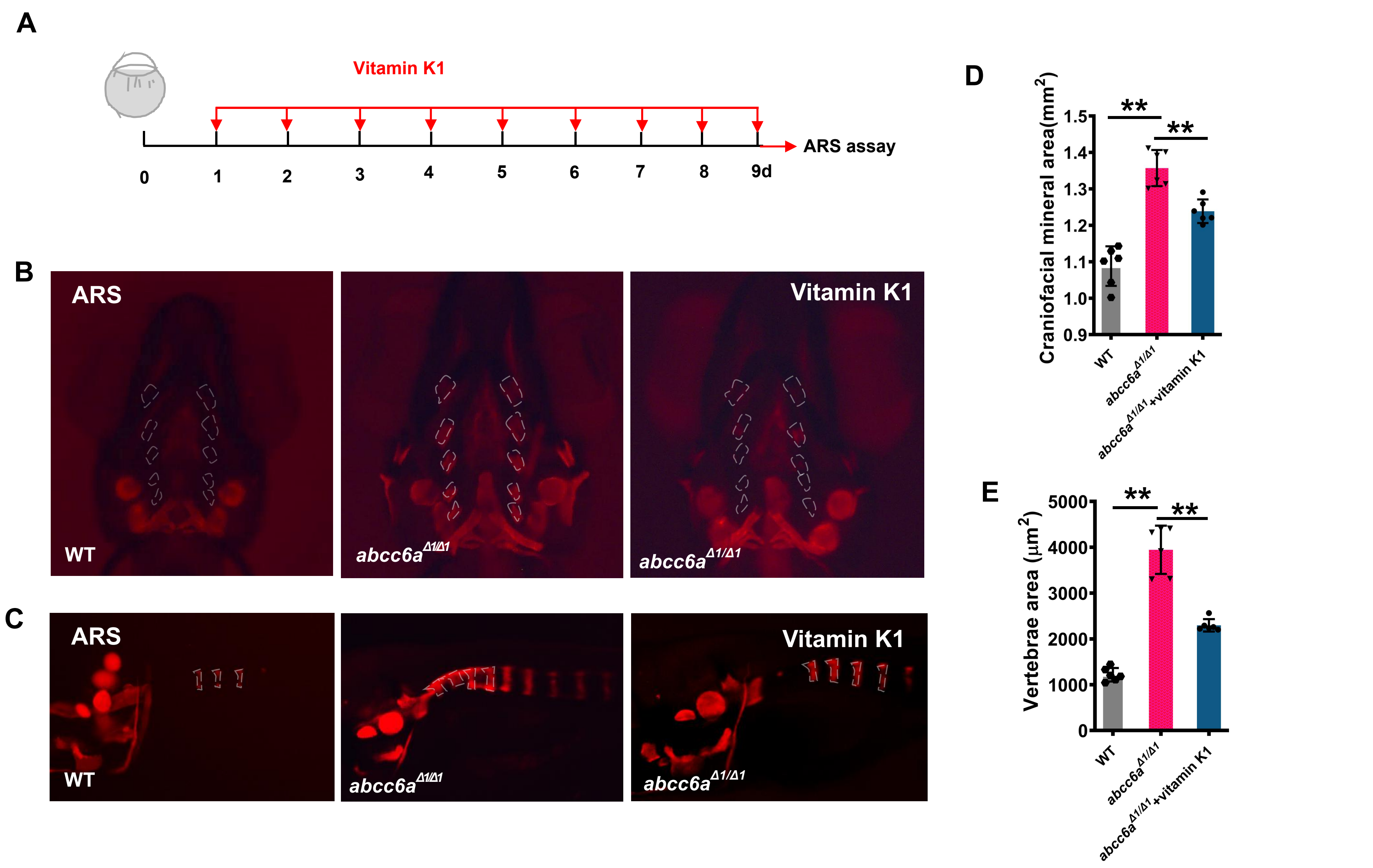


**Fig.S8 Vitamin K reduces craniofacial and vertebral hypermineralisation in *abcc6a* mutant embryos**

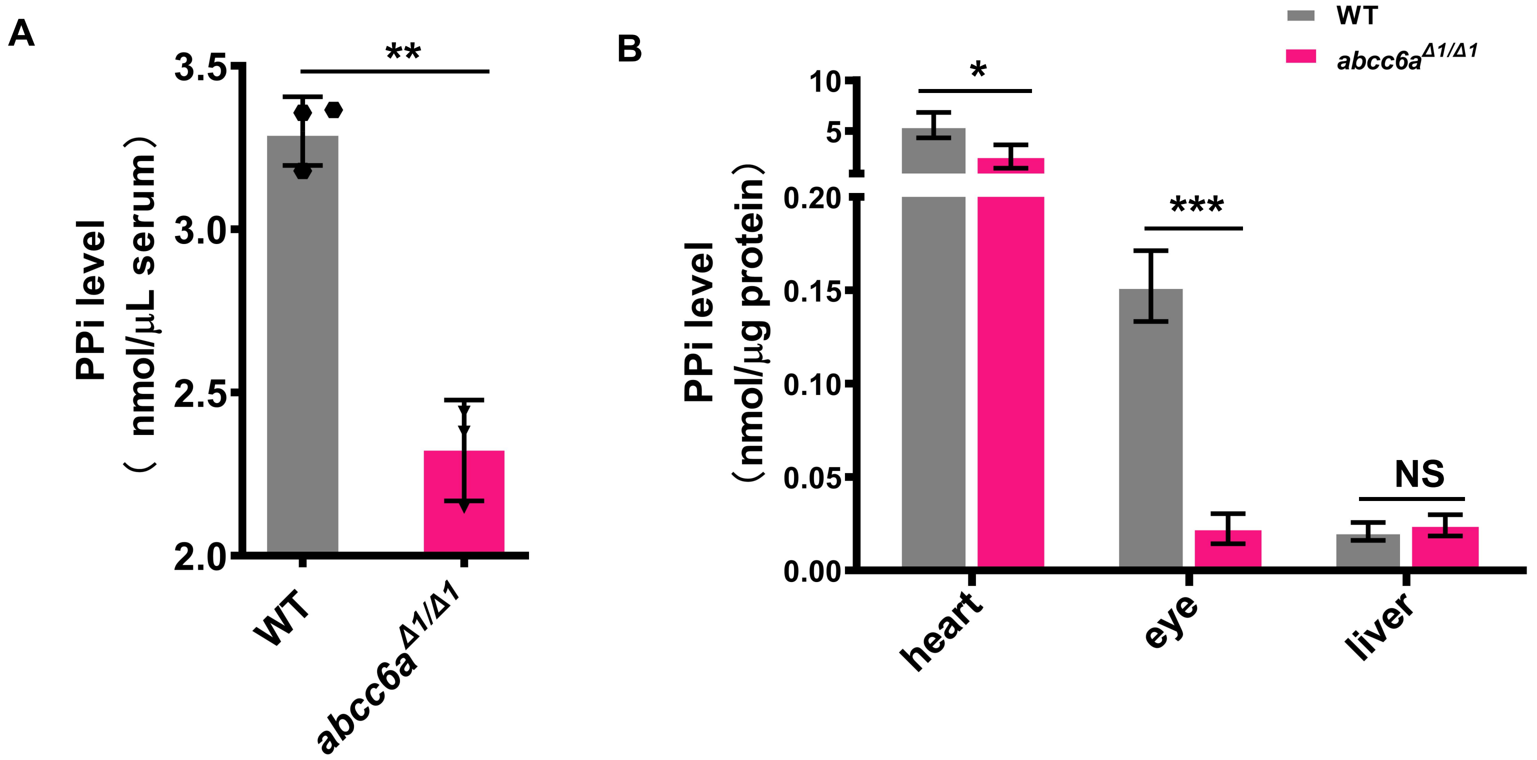


**Fig.S9 PPi levels in serum and tissue exhibit reductions in *abcc6a* mutant fish**
