# Supplementary material for: Disruption of Abcc6 Transporter in Zebrafish Causes Ocular Calcification and Cardiac Fibrosis
Source: Int J Mol Sci. 2020 Dec 29;22(1):278. doi: 10.3390/ijms22010278 (PMC7795442; doi:10.3390/ijms22010278)
Supplement: Supplementary file 1 [file ijms-22-00278-s001.zip › ijms-1002133-re-check-supplementary/Supplemental legends finalcheck.docx]

**Supplemental legends:**

Figure.S1 Kaplan-Meier survival curves of *abcc6a* mutantion. **(A)** Expression of *abcc6a, abcc6b.1*, *abcc6b.2* and *abcc1* were examined using qPCR analyses in WT and *abcc6a^Δ1/Δ1^* mutant hearts. *β-actin* expression was used for normalization. Data are mean ± SEM from 3 biological replicates. **(B)** Kaplan–Meier survival curves of WT and *abcc6a* mutant fish. Grey line represents WT group and red line represents *abcc6a^-/-^* group. Black dotted line represents half of the *abcc6a^-/-^* fish have died for 22 months. **(C-F)** Alizarin red staining showing the excessive calcification of the *abcc6a* mutants. (D, F) Higher-magnification images of the dashed boxes in (C, E). Brown arrow in the small image (F) indicates that ocular suborbital bones and supraorbital bones showed excessive calcification the *abcc6a* mutant eyes. Scale bar: 2mm (C, E); 1mm (D, F).

Figure.S2 *abcc6a^Δ2/Δ2^* mutants display vertebral hypermineralisation. **(A, B)** *abcc6a^Δ2/Δ2^* mutants exhibit malformed adult body axis curvature and short body length at 8 mpf. The red dotted line indicates the extent of the skull uplift. A small compartment represents 1 mm. **(C-F)** Micro-CT scan (C, E) and alizarin red staining (D, F) of WT and *abcc6a^Δ2/Δ2^* mutants showing vertebral hypermineralisation.

Figure.S3 Validation of ABCC6 antibody in several human and mice cell lines. **(A-D)** Human HepG2 cells derived from liver tissue (A, B) and mouse renal tubular epithelial cells (IMCD3) (C, D) were chose as the positive controls to verify and validate the ABCC6 antibody specificity. **(E-H)** RPE1 cells from human retinal pigment epithelium (E, F) and mouse chondrogenic cell (ATDC5) as the negative controls (G, H).

Figure.S4 *abcc6a* mutants display ocular fibrosis in adult eyes. **(A, B)** Abcc6 protein (green) recognized with anti-ABCC6 antibody overlaps with vascular endothelial cell marked by *flk:*mcherry (red). The immunofluorescence results showing that Abcc6 is located in the vascular-rich choroidal tissues by co-localization staining (yellow arrow) of *Tg (flk:mcherry)* fish. **(C-F)** Picrosirius red staining showing abnormal fibrotic accumulation in sclera of the *abcc6a* mutants. (D, F) Higher-magnification images of the dashed boxes in (C, E). Red arrow marks abnormal fibrosis of scleral layer in the *abcc6a^Δ1/Δ1^* mutant eyes. **(G, H)** Abcc6 immunostaining is detectable on the muscularis external and lamina propria with partial overlaps with smooth muscle actin antibody in the adult zebrafish intestine. Brown arrow in (G) point to some of enterocytes weakly expressing Abcc6. Insets (H) show higher-magnification images of the dashed box (G). **(I, J)** Picrosirius red staining revealing the abnormal increase in fiber content in the lamina propria structure in the *abcc6a^Δ1/Δ1^* mutant intestine. **(K)** Quantification of the width of the lamina propria from WT (n=15) or *abcc6a^Δ1/Δ1^* fish (n=19). ****P<0.0001, Student *t*-test (unpaired, two-tailed).

Figure.S5 *abcc6a^Δ2/Δ2^* mutants show serious heart malformations. **(A, B)** Whole-mounted photographs of adult heart at 8 mpf showing cardiac shrinkage and fibrosis phenotypes in *abcc6a^Δ2/Δ2^* (B), compared with WT sibling heart (A). **(C, D)** Hematoxylin-eosin (HE) staining exhibiting thinner compact layer and less myocardial cells in *abcc6a^Δ2/Δ2^* heart (D), compared to wild type sibling hearts (C). (E) Quantitative analysis showing thinner compact layer thickness in *abcc6a^Δ2/Δ2^* adult heart (n=13), compared with WT sibling heart (n=13). ***P<0.001, Student’s t-test (unpaired, two-tailed).

Figure.S6 *abcc6a* mutant hearts develop normally during embryonic and young adult stages. **(A, B)** In situ hybridization analysis showing that the expression of *cmlc2* in the *abcc6a^Δ1/Δ1^* mutant hearts (B) developed normally compared with the WT hearts (A). **(C, D)** Immunofluorescent section images of adult ventricles stained with anti-MF20 antibodies from WT (C) or *abcc6a^Δ1/Δ1^* mutant fish (D), showing that the density of cardiomyocytes was not affected. Scale bar: 100mm (A, B).

Figure.S7 *abcc6a* deficiency upregulates EMT genes. **(A, B)** WT and *abcc6a^Δ1/Δ1^* mutant ventricles were immunostained with anti-Collagen 1a (Col1a) antibody. **(C)** qPCR analyses of expression levels of *snail1a*, *snai1b*, *snai2/slug*, *snai3*, *mmp9* and *mmp13a* in WT hearts and *abcc6a* mutant hearts. *β-actin* expression was used for normalization. Data presents as mean ± SEM, n = 3, *P < 0.05, **P < 0.01, ***P < 0.001, Student *t*-test (unpaired, two-tailed). Scale bar: 20mm (A, B).

Figure.S8 Vitamin K reduces craniofacial and vertebral hypermineralisation in *abcc6a^Δ1/Δ1^* mutant embryos. **(A)** Schematic diagram of vitamin K1 treatment for rescue analysis. Red arrows represent experimental steps for vitamin K1 treatment. **(B, C)** Alizarin Red staining revealing a significant beneficial effect of vitamin K on craniofacial (B) and vertebral hypermineralisation (C). The dotted box represents the location of the focal calcification. **(D, E)** Quantification of craniofacial mineral area (D) and vertebrae area (E) showing that vitamin K reduces craniofacial and vertebral hypermineralisation in early *abcc6a^Δ1/Δ1^* mutants.

Figure.S9 PPi levels in serum and tissue exhibit reductions in abcc6a mutant fish. (A) (A-D) Graphical representation of pyrophosphate assay measurements for tissue and serum PPi levels in WT (n=9) and abcc6aΔ1/Δ1 fish (n=9) fish. *P < 0.05, **P < 0.01, ***P<0.001, Student’s t-test (unpaired, two-tailed). All experiments were performed in triplicate.

Supplementary Table S1 Primer sequences for qPCR amplification.

Supplementary Table S2 FPKM values per gene for AB and HO samples.
